# Effects of Tianshu Capsule on Spontaneously Hypertensive Rats as Revealed by ^1^H-NMR-Based Metabolic Profiling

**DOI:** 10.3389/fphar.2019.00989

**Published:** 2019-09-11

**Authors:** Jian Gao, Tieshan Wang, Chao Wang, Shuai Wang, Wei Wang, Di Ma, Yongbiao Li, Haibin Zhao, Jianxin Chen

**Affiliations:** ^1^The Third Affiliated Hospital, Beijing University of Chinese Medicine, Beijing, China; ^2^Beijing University of Chinese Medicine, Beijing, China; ^3^Dongfang Hospital, Beijing University of Chinese Medicine, Beijing, China; ^4^School of Life Science and Engineering, Southwest Jiaotong University, Chengdu, China

**Keywords:** Tianshu capsule, hypertensive, ^1^H-NMR, metabolism, SHR

## Abstract

Hypertension is one of the most common cardiovascular diseases, resulting in serious complications such as cardiovascular damage and chronic kidney disease. Tianshu capsule (TSC), composed of Chuanxiong (Ligusticum chuanxiong Hort) and Tianma (Gastrodiaelata Blume), has been widely used to treat the blood stasis type of headache and migraine in clinic. Results of previous research showed its antihypertensive effects, but the underlying mechanisms were still unclear. The purpose of this study was to evaluate the antihypertensive effect of TSC on spontaneously hypertensive rats by ^1^H NMR-based metabonomics and enzyme-linked immunosorbent assay (ELIAS), explore potential biomarkers and targets, and probe the potential mechanism of TSC on antihypertensive treatment. The results showed that TSC could decrease the product of oxidative stress (MDA) and enhance the activities of SOD and GSH-Px, down-regulate the expression of enzymes (LDHA, PKM2 and HK2) related to glycolysis, and perturb the levels of a series of amino acids (isoleucine, alanine, asparagine, citrate, etc.) and pathways. Multivariate statistical analyses showed remarkable changes in some endogenous metabolites after administrating TSC related to oxidative stress, amino acid metabolism and energy metabolism disturbances. Some enzymes (alanine-glyoxylate aminotransferase-2, tyrosine hydroxylase, dopa decarboxylase, etc.) related to metabolic biomarkers were predicted as the potential targets of TSC treatment on SHRs. The discoveries are helpful to understand the antihypertensive mechanism of TSC and provide theoretical evidence for its future research, development and clinical use.

## Introduction

Hypertension is the main cause of morbidity and mortality of cardiovascular diseases. It was described as one of the most common serous chronic diseases for global burden in 2013 (Collaborators et al., 2015). The cause of 90% of hypertension was still unclear as a result of its complex, multifactorial and polygenic character, and it was defined as essential hypertension (Oparil et al., 2003). Essential hypertension is a risk associated with development of cardio-cerebrovascular disease. Common treatment with anti-hypertensive medications could reduce the blood pressure and major cardiovascular event risks. Characterized with multiple levels, multiple compounds and comprehensive regulation, traditional Chinese medicine (TCM) is always composed of various herbs in one prescription and has been well accepted and applied in clinical settings in China to increase efficacy and attenuate toxicity (Schmidt et al., 2007). TCM has a long history for clinical use and could be one of the most effective sources for new drug development.

Tianshu Capsule (TSC) is a Chinese patent medicine approved by the China Food and Drug Administration. It is clinically used for headache caused by blood stasis and wind-heat syndrome with thousands of years of clinical experience (Commission, 2015). TSC is composed of Chuanxiong (*Ligusticum chuanxiong Hort*) and Tianma (*Gastrodiaelata Blume*) at a ratio of 4:1. It is reported that TSC has various effects on vertigo, cephalagra, rheumatoid arthritis pain, numbness in the limbs, neck stiffness and some problems relating to attention deficit and memory (Zhao et al., 2010). Taking a panoramic view of the domestic and foreign research status, the antihypertensive effects and underlying mechanisms of TSC are still unclear.

It is suggested that approximately 45% of hypertensive patients have metabolic syndrome (Akholkar et al., 2015), indicating the role of hypertension in dysregulation of energy balance. Metabolism could directly reflect the pathological state of hypertension. Various recent studies have reported the pathomechanism of essential hypertension (Carretero and Oparil, 2000; Doris and Fornage, 2005), while the perturbations in metabolites and biochemical pathways in hypertension and after drug exposure remain less explored. Metabolomics sensitively revealed what actually happens by collecting all metabolites and detecting metabolic responses to stimulation or intervention (Rochfort, 2005). Metabolomics is used here as a systems biology approach to explore the new pathophysiology of hypertension and elucidate the holistic molecular mechanisms of the action of drugs. Metabolic perturbations seen in hypertensive populations can be altered by drug treatment, which is a crucial mechanism in the antihypertensive effect of drugs. Drug treatments not only significantly rebalance the dynamics of metabolic fluxes but also elicit a network-wide reorganization of metabolism. The emerging application of metabolomics in drugs treating hypertension denote great potentialities in identifying new hypertensive biomarkers and establishing novel antihypertensive therapeutics.

Spontaneously hypertensive rats (SHR) are commonly used experimental animals at home and abroad in the research on prevention and treatment of human essential hypertension (Okamoto and Aoki, 1963; Pinto et al., 1998). Aa et al. analyzed the plasma samples of SHRs treated with total ginsenosides and further addressed the differential mechanism of total ginsenosides as compared with conventional drugs (Aa et al., 2010). ^1^H NMR-based metabolomics has been successfully applied to pharmacological and metabolism studies due to its inherent characteristics of no bias, high throughput and structural information richness. (Li et al., 2017). Feng et al. used ^1^H NMR-based metabolomics analysis to explore the mechanism of Qingre Huatan Decoction for treating hypertension in young male adults (Feng et al., 2015).

In our laboratory, ^1^H NMR-based serum metabonomics combined with multivariate data analysis methods was applied to explore the characteristic metabolic changes of SHR, complemented with enzyme-linked immunosorbent assay (ELISA), revealing that TSC’s effects on SHR rats and the result may enhance our cognition of the potential biochemical pathways in essential hypertension.

## Materials and Methods

### Chemicals and Drugs

TSC (license no. Z10950004) was purchased from Jiangsu Kanion Pharmaceutical Co., Ltd. (Batch No. 171206, Jiangsu, China). The chemical profiling of TSC has been reported in the literature (Liang et al., 2014). The SOD, MDA and GSH-px analysis kit were all purchased from Nanjing Jiancheng Bioengineering Institute (China).

### Experimental Animals

Male Sprague-Dawley (SD) rats, 40 male SHRs and 20 male WKYs weighing 230–250 g, were purchased from the Animal Breeding Center of Beijing Vital River Laboratories Company (Beijing, China). The spontaneously hypertensive rats were established by inbreeding the Wistar–Kyoto rat (WKY) with the highest blood pressure. The two strains have a similar genetic background, except for some hypertensive factors. Our protocol was fully in compliance with the standards of the Animal Ethical and Welfare Committee, Beijing University of Chinese Medicine.

The 30 SHRs and 10 WKYs were randomly divided into 4 groups (10 in each group): WKY-N group (distilled water), SHR-N group (distilled water), SHR-L group (0.43 g/kg, TSC) and SHR-H group (2.15 g/kg, TSC). During the administration period of 5 weeks, the SHR-H group and SHR-L group were given TSC intragastrically (i.g.). The WKY-N group and SHR-N group were scheduled for intragastric administration (i.g.) of distilled water. TSC was dissolved in distilled water. Each group received the same volume of distilled water or TSC solution (20 ml/kg) twice a day. After the rats became stabilized and the blood flow on the caudal artery was ensured by placing them on the holder with a heating table approximately at 37°C for 15 min, SBP and HR were measured with a non-invasive tail-cuff plethysmography method (BP-2006A, Softron, Beijing, China) once every week throughout the experiment (Chang et al., 2019).

### ^1^H NMR Spectroscopy and NMR Data Analysis

The ^1^H NMR spectra were all collected from a NMR system 600 NMR spectrometer (Varian Inc, Palo Alto, Calif), which operated at 599.808 MHz and was equipped with a 5 mm inverse-proton triple resonance probe. A one-dimensional NOESY-pre sat pulse sequence (RD-90°-t1-90°-tm-90°-ACQ) with a 2 s recycle delay and a 100 s mixing time was used.

^1^H NMR spectra of serum samples were all phase and baseline corrected using MestReNova software (version 7.1.0, Mestrelab Research, Spain). The chemical shift of TSP was referenced at δ0.00. Firstly, the water signals and related regions between 4.33 and 5.50 ppm were all excluded. Based on the code implemented in Matlab with an average of 0.015 ppm for each bin, all the NMR data were binned using an adaptive binning approach. To minimize the effects of unequal mass of each sample, probability quotient normalization was applied (Dieterle et al., 2006).

To attenuate the effects of dominant variables and noise while amplifying the weak signals as large as possible, the binned segments were subsequently mean centered and pareto-scaled prior to statistical analysis (Craig et al., 2006). “R” software was used for the univariate and multivariate analysis of the integral area of metabolites between groups. Multivariate statistical analysis could maximize the discrimination of both classes by filtering out irrelevant effects, including unsupervised principal component analysis (PCA) and supervised orthogonal projection to latent structure with discriminant analysis (OPLS-DA). The validity of OPLS-DA models with R2 (the total explained variation) and Q2 (the predictive capacity) was assessed *via* a repeated twofold cross-validation method and permutation test (n = 2,000).

A color-coded loadings plot was created to show the variables which could separate groups and mark the differential biomarkers. The variable importance in the projection (VIP) values of all peaks from OPLS-DA models was analyzed. The fold changes and the corresponding p-values of metabolites were calculated and corrected by the Benjamini and Hochberg method.

R software was used to calculate the data of metabolites. Based on the fold changes of all metabolites, the nodes were color coded. Red and blue separately suggested the increases and decreases of metabolites in emodin-treated groups versus the control group.

### Correlation Network Analysis

MetaboAnalyst 3.0 (http://www.metaboanalyst.ca/MetaboAnalyst/ ) and Cytoscape 3.7.1 were used for the metabolic pathway and metabolic correlation protein analysis. Pathways closely related to potential metabolic biomarkers were to explore and for further discussion. Proteins related to potential metabolic biomarkers were excavated with Cytoscape and the potential protein targets were verified in accordance with the literature.

### Determination of SOD, MDA and GSH-px in the Serum

After treatment, rat blood samples were obtained from the abdominal aorta, and serum samples were then separated by centrifugation at 1,000 g, 4°C for 10 min. The activity of glutathione peroxidase (GSH-Px) and superoxide dismutase (SOD) and the concentration of malondialdehyde (MDA) were determined as an index of lipid peroxidation in serum samples using the spectrophotometric method as described in the commercial kits (Nanjing Jiancheng Bioengineering Institute, China) according to the manufacturer’s instructions.

### ELISA (LDHA, PKM2 and HK2)

The primary function of pyruvate kinase isoform M2 (PKM2) is to catalyze the phosphorylation from phosphoenolpyruvate to pyruvate as the last step of glycolysis to generate ATP. The lactate dehydrogenase-A (LDHA) is an enzyme that converts pyruvate into lactate. The hexokinase 2 (HK2) is an enzyme responsible for glucose’s commitment to the glycolytic pathway through the phosphorylation of glucose. Serum samples of each group were isolated and collected for ELISA analysis. HK2, PKM2 and LDHA concentrations were detected using ELISA kits (Cloud-Cone Company, Wuhan, China) according to the instructions in the commercial kits.

### Statistical Analysis

Statistical analysis was performed with GraphPad Prism 5.0 software (GraphPad Software, Inc, La Jolla, CA, USA). All data were expressed as mean ± SD. The statistical tests were one-way ANOVA followed by post-hoc Newman-Keuls multiple comparisons test. A probability level of 0.05 was considered statistically significant.

## Results

### Chemical Constituents in TSC and Effect of TSC on Systolic Blood Pressure (SBP) and Heart Rate in Non-Anesthetized WKYs and SHRS

TSC is composed of *Ligusticum chuanxiong Hort* (Chuanxiong in China) and *Gastrodia elata Blume* (Tianma in China) with a crude weight ratio of 4:1. Chemical constituent analysis of TSC showed that a total of 38 compounds were identified or tentatively characterized (Liang et al., 2014). Organic acids, phenols, phthalides, and nitrogen-containing compounds were the major active ingredients of TSC, which included gastrodin, parishin B/C/E/G, ferulic acid, senkyunolide A/H/I, ligustilide, neocnidilide, 3-butylidenephthalide, etc. The results of the literature research showed that ferulic acid is the major active ingredient in *Ligusticum chuanxiong Hort*, and it has been chosen as the marker component for controlling the quality of *Ligusticum chuanxiong Hort* in Chinese Pharmacopeia (Xia et al., 2013). Pharmacological studies have shown that ferulic acid possess anti-oxidant, anti-inflammatory, anti-thrombus and cardiovascular protection, free radical scavenging and blood pressure-lowering effects (Barone et al., 2009; Zhang et al., 2010; Commission, 2015). Gastrodin is the primary active constituent in *Gastrodia elata Blume*, and it has been selected as the marker component for controlling the quality of *Gastrodia elata Blume* in Chinese Pharmacopeia (Wang et al., 2007; Guan et al., 2017). Gastrodin has been reported to exhibit cardiovascular and cerebrovascular protection as well as sedative, anti-convulsive, free radical scavenging and analgesic activities (Li et al., 2006; Commission, 2015). Liang et al. carried out cell experiments and drew the conclusion that gastrodin, 5-HMF and ferulic acid in TSC may be the material basis of Tianshu capsules for the treatment of diseases caused by blood stasis (Liang et al., 2013).

Effects of the TSC on SBP and heart rate of SHRs and WKYs have been reported in previous research (Chang et al., 2019). Chang et al. reported that TSC significantly reduced the SBP at doses of 2.15 g/kg/day compared to SHR-Ns (*p* < 0.05). However, there was no significant difference in SBP of WKYs or heart rate in any group throughout the experiment.

### ^1^H NMR Analysis of Serum Sample and Multivariate Analyses of ^1^H NMR Spectra

**Figure 1:**
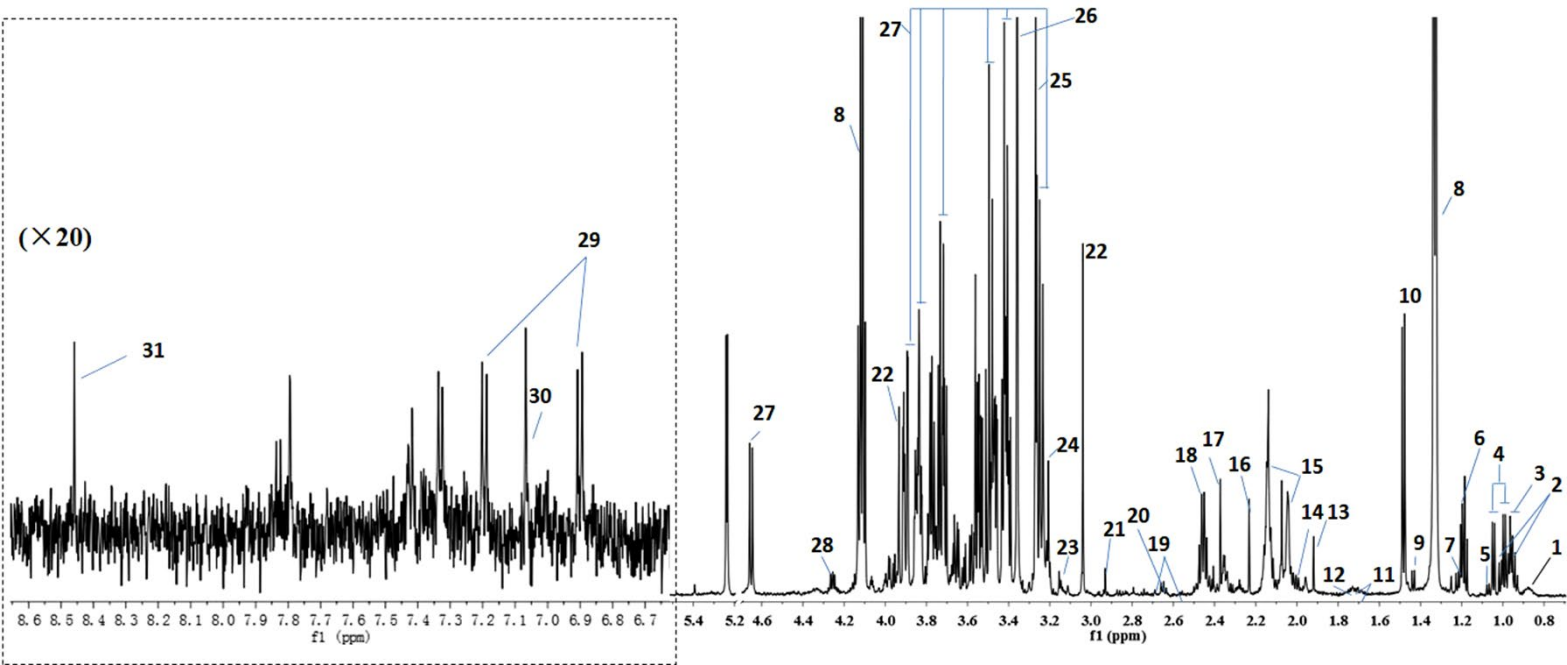
Shows representative ^1^H-NMR spectra with peak assignments for serum sample from SHR-N group. 1. VLDL/LDL, 2. Isoleucine, 3. Leucine, 4. Valine, 5. Isobutyrate, 6. 3-Hydroxybutyrate, 7. 3-Aminoisobutyrate, 8. Lactate, 9. Lysine, 10. Alanine, 11. Arginine, 12. Lysine, 13. Acetate, 14. Proline, 15. N-acetylated glycoprotein (NAC), 16. Acetone, 17. Pyruvate, 18.Glutamine, 19. Citrate, 20. Methionine, 21. N,N-Dimethylglycine, 22. Creatine, 23.Methylristidine, 24. Choline, 25. Betaine, 26. Methanol, 27. Glucose, 28. Threonine, 29. Tyrosine, 30. Methylistidine, 31. Formate.

^1^H NMR analysis was used to detect the change of substances in the serum sample. **Figure 1** shows representative ^1^H-NMR spectra with peak distribution of serum sample from the SHR-N group. The spectra illustrated the majority of metabolites, and metabolites were identified according to in-house NMR database, previous literature (Carrola et al., 2016; Liu et al., 2016) and the Human Metabolome Database.

**Figure 2:**
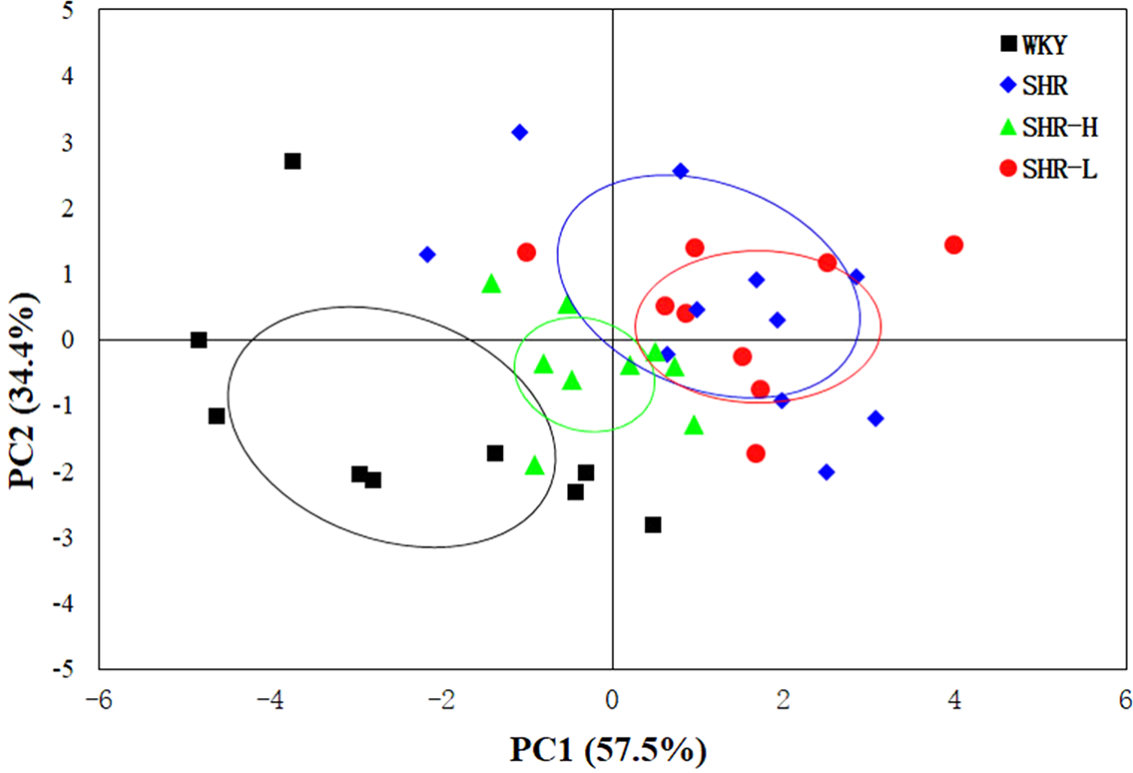
The PCA score plot for ^1^H NMR data showed a clear separation between the WKY-N and SHR-N groups along PC1.

PCA on the serum data from different pair-wise groups were to reveal distribution and show clusters of the whole subjects. The PCA score plot (**Figure 2**) showed a visible separation between WKY-N and SHR-N, while the SHR-H and TSC dose groups showed some overlaps with each other, with values of PC1 = 57.5% and PC2 = 34.4% in the serum samples. It was indicated that TSC induced the metabolic variations in rats.

**Figure 3:**
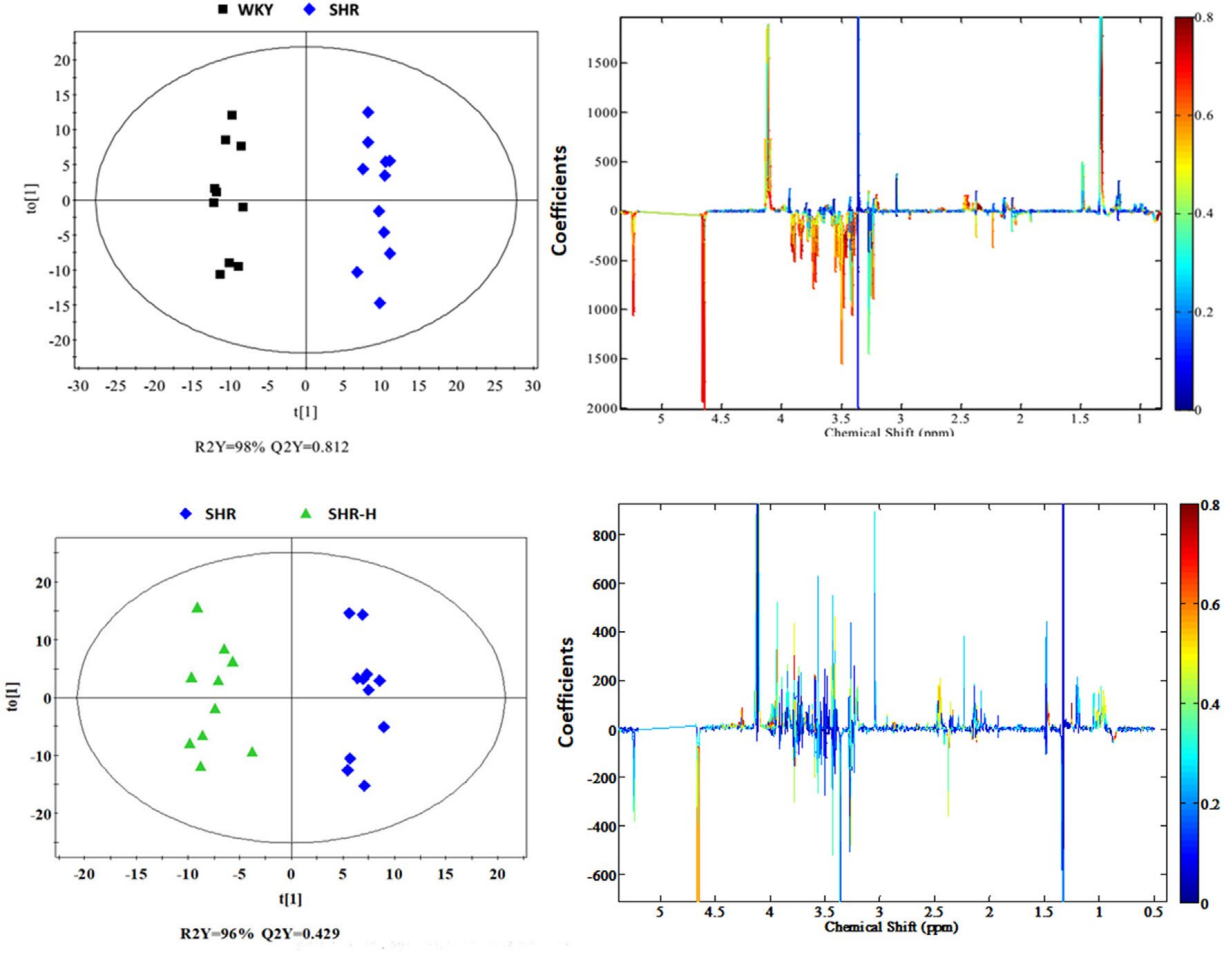
OPLS-DA score plots (left panel) and corresponding coefficient loading plots (right panel) derived from ^1^H NMR spectra of serum samples from the WKY, SHR and SHR-H groups.

To explore the metabolites which induced the separation, the OPLS-DA score plots and corresponding loading plots were generated from these three groups (**Figure 3**). The parameters from the WKY-N and SHR-N groups of the serum sample were R2Y = 98%, Q2Y = 0.812, and from the SHR-N and SHR-H groups of the serum sample they were R2X = 96%, Q2Y = 0.429 in the OPLS-DA model, presenting the variance and high predictive capability, respectively (Westerhuis et al., 2010; Massimi et al., 2012; Liu et al., 2014).

The color-coded coefficient plots were demonstrated to reveal the changes of metabolites in SHR-N rats or after TSC treatment. In the plots, the metabolites were characterized by significant changes (p < 0.05) from SHR-N rats relative to the WKY-N group or treatment rats relative to the WKY-N group.

The most vital biomarkers contributing to the separation of OPLS-DA were extracted as follows: increased consumption of 3-aminoisobutyrate, 3-hydroxybutyrate, alanine, asparagine, aspartate, choline, fumarate, glutamate, glutamine, lactate, methionine, *myo*-Inositol, pyruvate, serine, threonine, tyrosine and valine, together with reduced excretion of Isoleucine, betaine, cytidine, formate, glucose, glycerol, glycine, LDL/VLDL, Lipids, lysine and N,N-dimethylglycine were found in the serum of the SHR-N group compared to WKY-N. The 28 significantly changed metabolites (chemical shift, fold change, VIP value, *p* value, etc.) between the SHR-N and WKY-N groups are summarized in **Table 1**.

**Table 1:** The identified metabolites from NMR spectra of the control and SHR-N rats’ serum samples.

Metabolites	Chemical shift (ppm)(multiplicity)	*p*^a^	VIP	FC^b^
Isoleucine	0.94(t);1.01(d)	0.00	1.81	0.82
3-aminoisobutyrate	1.23(d)	0.01	1.60	1.15
3-Hydroxybutyrate	2.31(m); 2.41(m); 4.16(m)	0.00	2.01	1.36
Alanine	1.48(d)	0.00	2.00	1.18
Asparagine	2.87(m); 2.96(m)	0.03	1.36	1.22
Aspartate	2.69(s)	0.00	2.10	1.40
Betaine	3.27(s); 3.91(s)	0.00	1.98	0.85
choline	3.20(s)	0.00	1.98	3.20
cytidine	6.07(d)	0.00	1.77	0.44
formate	8.46(s)	0.02	1.41	0.83
Fumarate	6.53(s)	0.02	1.37	1.70
glucose	3.42(t); 3.54(dd); 3.72(t); 3.74(m); 3.84(m); 5.24(d)	0.00	2.01	0.78
glutamate	2.08(m); 2.15(m);2.35(m)	0.00	2.50	1.54
glutamine	2.14(m); 2.45(m); 3.80(m)	0.00	1.82	1.21
glycerol	3.58(m); 3.66(m); 3.80(m)	0.04	1.27	0.87
glycine	3.56(s)	0.00	2.06	0.84
lactate	1.33(s); 4.11(q)	0.00	1.69	1.32
LDL/VLDL	0.89(br); 1.31(br); 1.57(br)	0.01	1.52	0.56
Lipids	2.10(br); 2.24(br),5.31(br)	0.00	1.71	0.90
Lysine	1.86(m); 1.88(m); 3.03(t); 3.76(t)	0.02	1.39	0.74
methionine	2.14(s); 2.64(t)	0.00	2.08	1.24
myo-Inositol	3.28(t); 3.63(t); 4.07(t)	0.00	2.32	1.25
pyruvate	2.37(s)	0.00	2.58	1.45
serine	3.83(m); 3.98(m)	0.01	1.66	1.13
threonine	4.28(m)	0.03	1.35	1.21
N,N-dimethylglycine	2.93(s)	0.00	1.72	0.76
tyrosine	6.89(d); 7.19(d)	0.00	1.82	1.29
valine	0.99(d); 1.04(d); 2.27(m)	0.047	1.23	1.09

a The p values were obtained from student’s t-test. The chemical shifts in boldface were what we used in calculating integrals and p values.

b FC, Fold change values; the increased and the decreased in each group.

There were 20 significantly changed metabolites (chemical shift, fold change, VIP value, *p* value etc.) between the SHR-N and SHR-H groups, which are summarized in **Table 2**. Increased consumption of glucose, LDL/VLDL and pyruvate, together with reduced excretion of isoleucine, alanine, asparagine, citrate, creatine, glutamate, glutamine, histidine, lactate, lysine, methionine, *myo*-Inositol, serine, threonine, N,N-dimethylglycine, tyrosine and valine were found in the serum of the SHR-H group compared to SHR-N.

**Table 2:** The identified metabolites from NMR spectra of the SHR-H and SHR-N rats’ serum samples.

Metabolites	Chemical shift (ppm)(multiplicity)	*p*^a^	VIP	FC^b^
Isoleucine	0.94(t);1.01(d)	0.01	2.08	0.88
alanine	1.48(d)	0.00	2.64	0.71
Asparagine	2.87(m); 2.96(m)	0.02	1.91	0.88
citrate	2.56(d); 2.67(d)	0.01	2.14	0.74
creatine	3.04(s); 3.93(s)	0.01	2.25	0.86
glucose	3.42(t); 3.54(dd); 3.72(t); 3.74(m); 3.84(m); 5.24(d)	0.00	2.40	1.55
glutamate	2.08(m); 2.15(m);2.35(m)	0.02	1.91	0.88
glutamine	2.14(m); 2.45(m); 3.80(m)	0.02	1.92	0.87
histidine	3.99(m); 7.07(m)	0.02	1.91	0.76
lactate	1.33(s); 4.11(q)	0.03	1.77	0.81
LDL/VLDL	0.89(br); 1.31(br); 1.57(br)	0.00	2.49	1.50
Lysine	1.86(m); 1.88(m); 3.03(t); 3.76(t)	0.04	1.75	0.85
methionine	2.14(s); 2.64(t)	0.00	2.56	0.86
*myo*-Inositol	3.28(t); 3.63(t); 4.07(t)	0.00	2.37	0.88
pyruvate	2.37(s)	0.00	2.46	1.24
serine	3.83(m); 3.98(m)	0.00	2.27	0.89
threonine	4.28(m)	0.00	2.41	0.67
N,N-dimethylglycine	2.93(s)	0.00	1.76	0.80
tyrosine	6.89(d); 7.19(d)	0.00	2.35	0.79
valine	0.99(d); 1.04(d); 2.27(m)	0.01	2.09	0.99

a The p values were obtained from student’s t-test. The chemical shifts in boldface were what we used in calculating integrals and p values.

b FC, Fold change values; the increased and the decreased in each group.

The 13 metabolites (glucose, LDL/VLDL, alanine, asparagine, glutamate, glutamine, lactate, methionine, *myo*-Inositol, serine, threonine, tyrosine, valine), which changed significantly both in SHR-N rats and after treatment with TSC, were considered to be potential biomarkers.

### Metabolic Pathway Analysis and Metabolic Correlation Protein Analysis

Potential biomarkers are the same variations which were extracted from SHR-N vs WXY-N and TSC vs SHR-N, based on the multiple analyses above. MetaboAnalyst 3.0 was subsequently used to find the relevant metabolic pathways response to the acquired potential metabolic biomarkers (Xia and Wishart, 2016).

**Figure 4:**
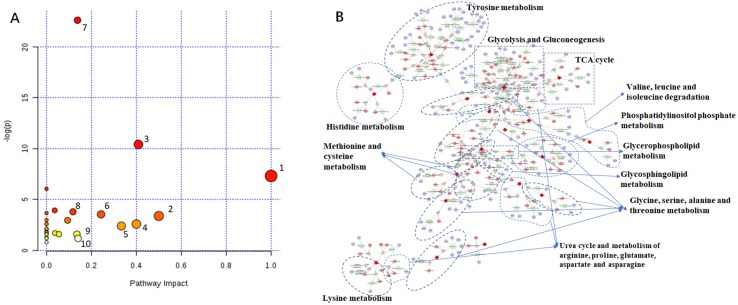
Metabolic Pathway Analysis of TSC-treated rats. **(A)** 1. Aminoacyl-tRNA biosynthesis, 2. Alanine, aspartate and glutamate metabolism, 3. D-Glutamine and D-glutamate metabolism, 4. Cysteine and methionine metabolism, 5. Glycine, serine and threonine metabolism, 6. Phenylalanine, tyrosine and tryptophan biosynthesis, 7. Methane metabolism, 8. Valine, leucine and isoleucine biosynthesis, 9. Inositol phosphate metabolism, 10. Tyrosine metabolism. **(B)** Red hexagon: metabolites; green round rectangle: enzyme; blue elipse: protein; gray diamond: reaction.

A different point stood for a different metabolic pathway, and the size of points in **Figure 4A** represented the impact value that was calculated from the pathway topology analysis. Pathways with an impact value above 0.1 were screened out as potential target pathways. There were 10 metabolic pathways in total involved in this study, including aminoacyl-tRNA biosynthesis, alanine, aspartate and glutamate metabolism, D-glutamine and D-glutamate metabolism, cysteine and methionine metabolism, glycine, serine and threonine metabolism, phenylalanine, tyrosine and tryptophan biosynthesis, methane metabolism, valine, leucine and isoleucine biosynthesis, inositol phosphate metabolism and tyrosine metabolism. As shown in **Figure 4B**, the metabolic correlation gene analysis was generated and drawn by Cytoscape. Pathways generated by Cytoscape were labelled in the figure and mainly involved the same amino acid metabolism and energy metabolism. With these results, key proteins related to the metabolic biomarkers were discovered.

**Figure 5:**
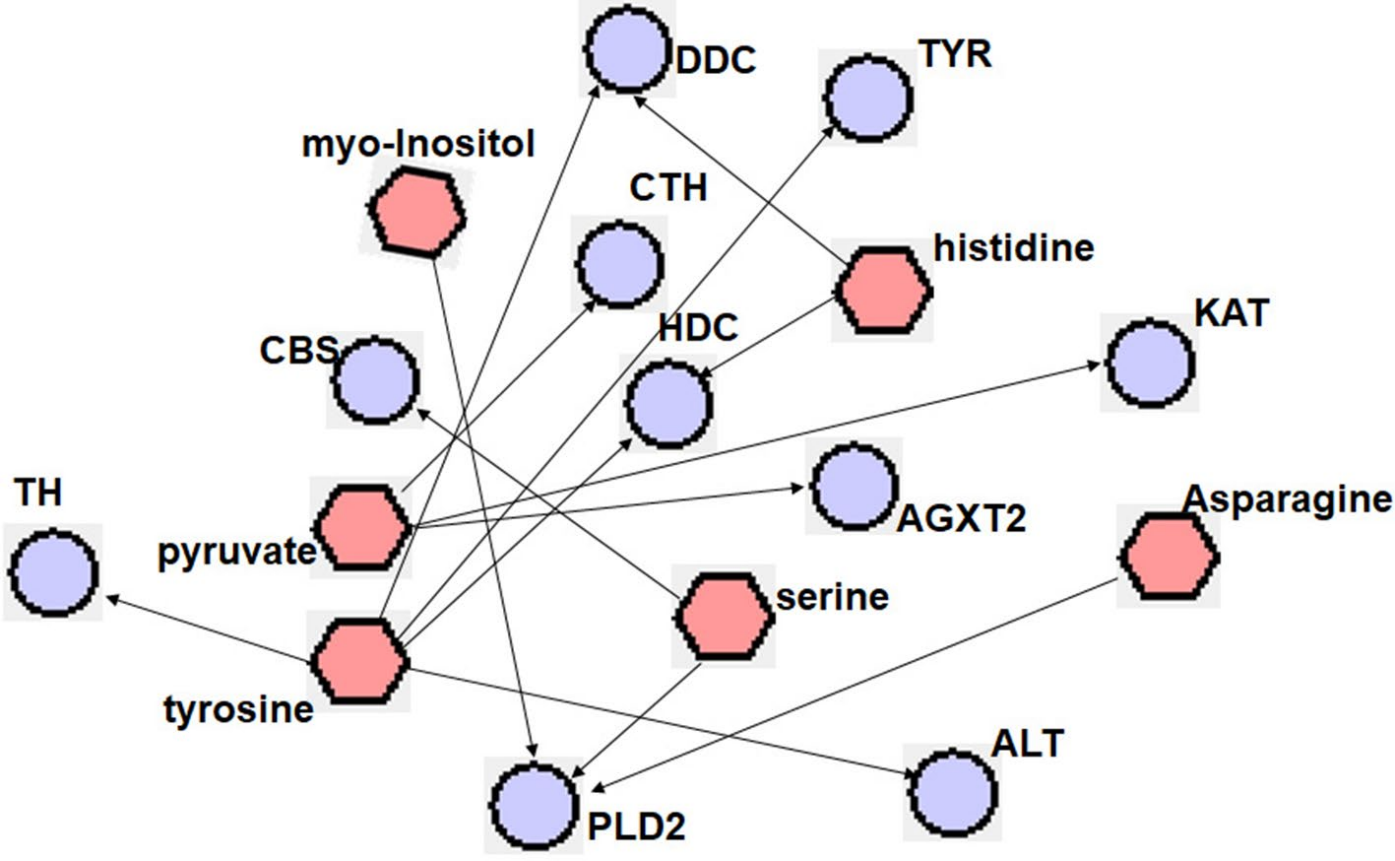
Metabolites associated with hypertension-related proteins. Metabolites and proteins are represented with 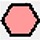 and 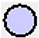, respectively. TH, tyrosine hydroxylase; CBS, cystathionine-beta-synthase; DDC, dopa decarboxylase; CTH, cystathionase (cystathionine gamma-lyase); TYR, tyrosinase; HDC, histidine decarboxylase; PLD2, b phospholipase D2; AGXT2, alanine-glyoxylate aminotransferase 2; KAT, kynurenine aminotransferase; ALT, gaspartate aminotransferase 2.

With the exploitation of metabolite-related proteins and researching the literature (Schroeder and Adams, 1941; Masserano and Weiner, 1983; Yoshimura et al., 1987; Ito et al., 2000; Burkhard et al., 2001; Kwok et al., 2002; Whitfield et al., 2002; Nakanishi et al., 2004; Han et al., 2010; Kimura, 2011; Caplin et al., 2012; Congo Carbajosa et al., 2015), finally 10 proteins (TH, CBS, DDC, CTH, TYR, HDC, PLD2, AGXT2, KAT, ALT) considered as potential markers of TSC on SHR rats were preliminarily confirmed. Metabolite-protein correlations are listed in **Figure 5**.

### Inhibited Glycolysis Pathway

Lactate is a marker of glycolysis, and the disturbance of lactate indicated that glycolysis alterations might have been involved in the anti-hypertensive effects of TSC. Results of the metabolomics analysis showed that lactate was significantly increased in SHR-N rats and decreased after exposure to TSC (**Tables 1** and **2**). Increased lactate levels in the SHR-N rats suggested a shift of energy metabolism toward anaerobic glycolysis during hypertension. The role of TSC in the glycolysis pathway was further investigated with ELISA to evaluate the glycolysis-related protein concentrations of rats in the WKY-N, SHR-N, SHR-H and SHR-L groups.

**Figure 6:**
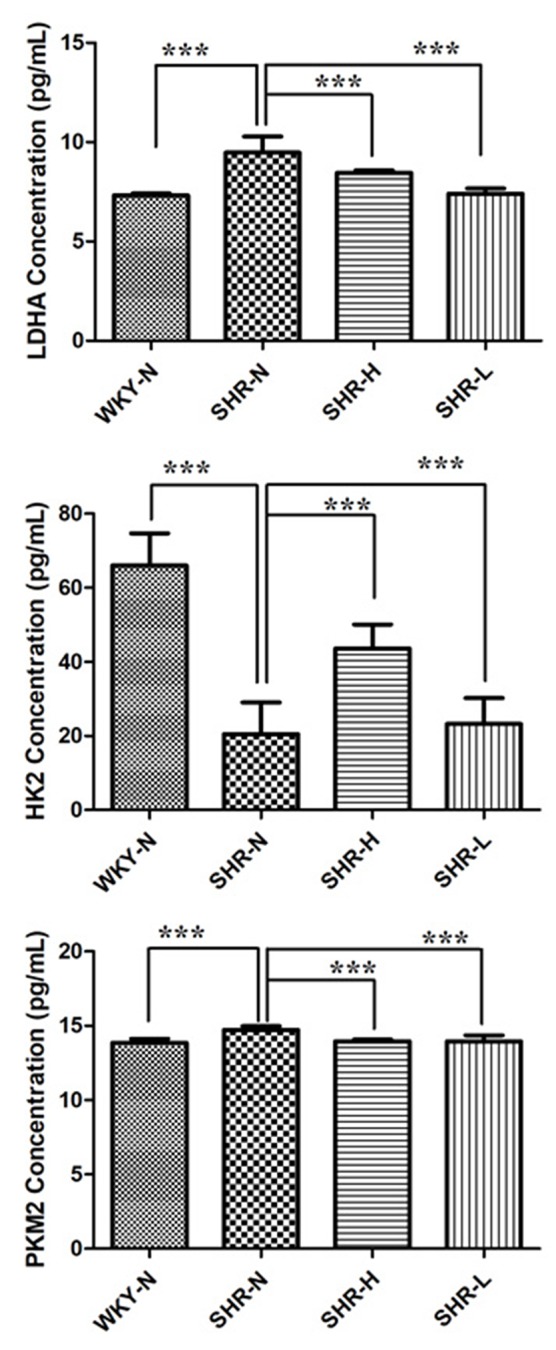
Expressions of glycolysis-related enzymes after treatment with TSC. Data are expressed as the mean ± SD and were analyzed by ANOVA. ***p < 0.0001.

The concentrations of HK2, PKM2 and LDHA were increased in SHR-N rats and were all decreased in the serum of TSC treated rats (SHR-L groups, p < 0.05) (**Figure 6**). The results demonstrated that the glycolysis pathway enhanced during the hypertensive process and TSC may have inhibited glycolysis of SHR.

### Inhibited Oxidative Stress

**Figure 7:**
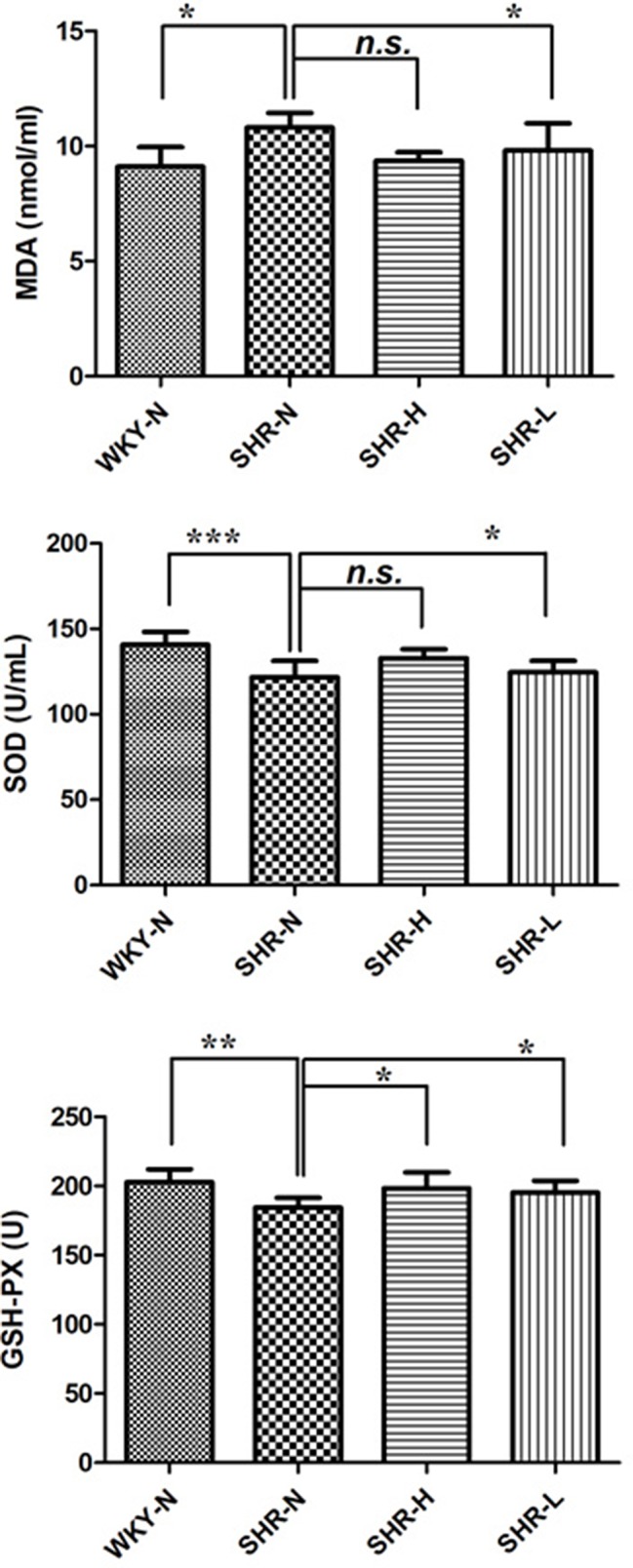
Expressions of SOD, MDA and GSH-Px after treatment with TSC. Data are expressed as the mean ± SD and were analyzed by ANOVA. ***p < 0.0001, **p < 0.001, *p < 0.01, n.s.: p > 0.05.

Oxidative stress played a role in the pathogenesis of hypertension, while hypertension itself could also contribute to oxidative stress (Montezano and Touyz, 2012). The levels of malondialdehyde (MDA), superoxide dismutase (SOD), and glutathione peroxides (GSH-Px) were usually used to display the oxidative and antioxidative status, respectively (**Figure 7**). The ELISA results showed that the MDA levels got higher in SHR-N vs WKY-N, while SOD and GSH-Px activities were lower in SHR-N vs WKY-N (*p* < 0.05). Compared with their levels in the model group, the levels of MDA in the TSC treated groups (SHR-H) were significantly decreased (*p* < 0.05), and SOD and GSH-Px activities in TSC-treated groups (SHR-H) were all increased (*p* < 0.05).

## Discussion

Hypertension is a chronic and complex disease related to systemic multiple systems, such as the central nervous system, peripheral nervous system, endothelial system, and kidneys. A variety of factors may be involved in the causes of hypertension, such as the interaction of environment and heredity factors leading to disturbances of blood pressure regulation, the pathological mechanism of which includes the sympathetic nervous system, renin-angiotensin-aldosterone system (RAAS), vasopressin, nitric oxide (NO), endothelin (ET), and a variety of vasoactive peptides secreted by other endothelial cells and smooth muscle cells (Sakao et al., 2009; Berger et al., 2009). From the perspective of Chinese Medicine, the pathogenesis of hypertension is considered as being founded on yin deficiency, with yang hyperactivity in the superficiality and phlegm-dampness and blood stasis penetrating all along; therefore, the basic therapeutic methods should be to supplement qi and nourish yin (Xu and Chen, 2007). The common clinical manifestations of hypertension are dizziness, headache, fatigue, lassitude in the loins and knees, and so on.

TSC, a proprietary Chinese medicine, consists of *Gastrodia elata* (Tianma) and *Ligusticum chuanxiong Hort* (Chuanxiong). Tianma could calm the liver and suppress liver yang hyperactivity. The indications of Tianma are dizziness, tinnitus, distending feeling in head, headache, facial flushing and conjunctival congestion, the pathogenesis of which belongs to flaming up of liver fire and hyperactivity of liver yang. Chuanxiong could promote qi and activate blood circulation to relieve pain. According to documentation in Shengnong Ben Cao Jing (Shennong’s Classic of Meteria Medica), it can treat headache (Wang and Xiong, 2012).

In the current hypertension research, SHRs have mostly been used in animal models, as their pathophysiological processes were similar to those of essential hypertension (Pinto et al., 1998). In metabolomics, NMR is the relative analytical robustness and reproducibility of its great strength compared to MS. In this study, we introduced a ^1^H NMR-based metabolomics approach to dig up the potential biomarker-related affected metabolic pathways and proteins in TSC-administrated SHRs. The above results revealed that TSC intervened in the process of energy metabolism, amino acid metabolism, and even oxidative stress during the treatment of hypertension. And 10 proteins (TH, CBS, DDC, CTH, TYR, HDC, PLD2, AGXT2, KAT and ALT) were considered as potential markers of TSD on SHR rats and may attend the metabolomic processes.

### Energy Metabolism

**Figure 8:**
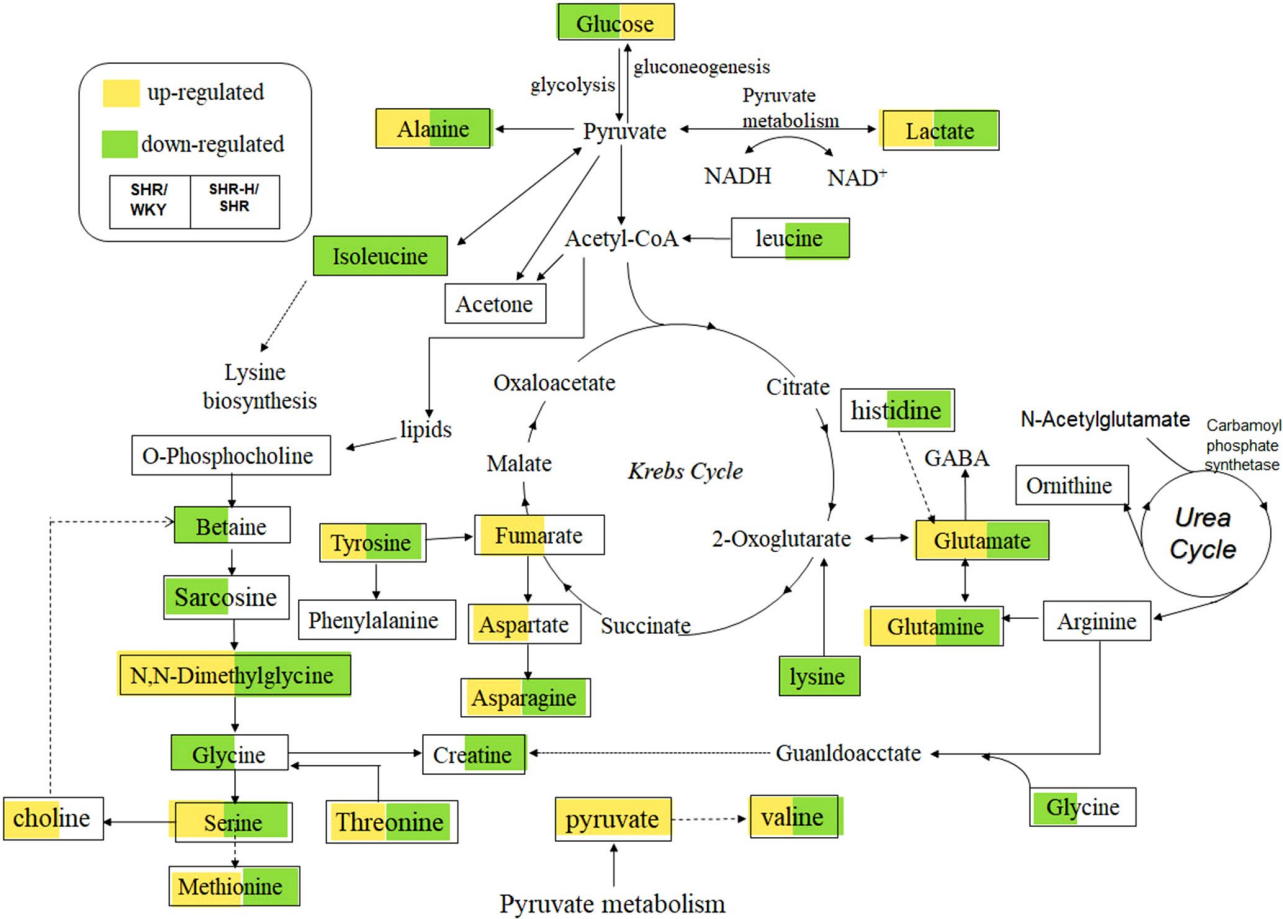
Perturbed metabolic pathways detected by ^1^H NMR analysis.

The metabolomics results revealed that lactate was remarkably increased in SHR rats and decreased after being exposed to TSC (**Figure 8**, **Tables 1** and **2**). Kazuki Akira et al. (2013) and Matsutomo et al. (2017) both found the plasma level of betaine changed remarkably in the SHRs. Betaine is endogenously produced from choline and its concentration in human plasma and urine is controlled by the homeostatic system. Lactate is a marker of glycolysis, and glycolysis alterations might be closely associated with the anti-hypertensive effects of TSC (Xing et al., 2018). As a critical substrate for energy metabolism, pyruvate is mainly derived from glycolysis and catabolism of certain amino acids (Li et al., 2014). In our results, there was a decrease in glucose level; however, increased levels in lactate in the SHR-N rats suggested an up-regulation in both mitochondrial glucose oxidation and glycolytic metabolism. A sharp increase in both lactate and pyruvate may indicate a shift of energy metabolism toward anaerobic glycolysis during the hypertension.

Glucose level reduced in SHR-N rats, suggesting an excess consumption of glucose as energy substrates, which leads to a deficiency of glucose in peripheral blood. Additionally, lactate level increasing may indicate the overall metabolic shift to favor the production of ATP *via* glycolysis at the expense of oxidative phosphorylation in response to hypoxia even with adequate oxygen, known as the Warburg Effect, which was firstly described in tumor cells (Zhao et al., 2013). Our data showed a tendency to bring the levels of glucose, lactate and pyruvate to normal with TSC treatment in SHR-N rats, which may indicate that TSC disturbed the glycolysis on treatment of hypertension, thus its potential effect on energy mechanisms deserves further exploration.

### Amino Acid Metabolism

A decrease in amino acids isoleucine, glycine and lysine, and an increase in alanine, asparagine, aspartate, methionine, serine, threonine, tyrosine and valine were detected in the SHR-N group (**Figure 7**). The metabolite significantly altered by the TSC treatment was associated with D-Glutamine and D-glutamate metabolism, alanine, aspartate and glutamate metabolism, phenylalanine, tyrosine and tryptophan biosynthesis, tyrosine metabolism, alanine, leucine and isoleucine biosynthesis, glycine, serine and threonine metabolism.

Dietrich et al. indicated that metabolic alterations of serine and glycine in serum occurred early in the development of hypertension (Dietrich et al., 2016). Anti-inflammatory and antioxidant properties were attributed to serine and glycine, which may contribute to protective effects regarding the development of hypertension. Aa et al. identified 3-hydroxybutyrate, glucose, glutamate, pyruvate, serine and threonine as potential markers, which differed significantly between SHRs and WKYs (Aa et al., 2010). The results were consistent with ours, and these metabolites were identified as the biomarkers of hypertension.

Alanine was reported to reduce the release of nor-epinephrine from cardiac sympathetic nerves in animal models and finally increased the blood pressure (Butta and Adler-Graschinsky, 1987). Alanine played roles in blood pressure elevation, which seemed associated with the catecholamines’ modulation of cardiovascular responses (Conlay et al., 1990). In addition, the interaction between alanine and insulin also affected arterial blood pressure, which could be explained as the consumption of alanine leading to the release of insulin, thereby the level of insulin affected the blood pressure (Newsholme et al., 2005). Ben Caplin et al. (Caplin et al., 2012) provided strong evidence that alanine-glyoxylate aminotransferase-2 (AGXT2) metabolizes endogenous methylarginines *in vivo*, and disruption of this enzyme also leads to reduced NO bioavailability and hypertension.

Evidence suggests that SHRs are much more sensitive to the excitatory and excitotoxic effects of glutamate (Kapoor et al., 1994). The levels of glutamate and glutamine were significantly increased in SHRs and decreased after TSC treatment, while the ratio of glutamate/glutamine declined after TSC administration, which showed that the enhanced glutaminase activity converted more glutamine to glutamate. As a result, glutamate’s amino acid product, alanine, was markedly decreased. Kynurenic acid is an excitatory amino acid (such as glutamate and aspartate) antagonist. SHR is associated with abnormally low kynurenic acid levels in the area of CNS which controls physiological blood pressure (Ito et al., 2000; Kwok et al., 2002), while the aminotransferase, capable of catalyzing the transamination of kynurenine to kynurenic acid (KYNA) using various co-substrates, has commonly been termed kynurenine aminotransferase (KAT) (Han et al., 2010). KAT was firstly reported to be associated with the treatment of TSC on hypertension, but the potential mechanism needs to be further explored.

It was reported that asparagine could be converted to ornithine and produce urea participating in the urea cycle (Moncada and Higgs, 1993). Our data showed there was an increasing tendency of asparagine level and this may be interpreted as the urea metabolic disorders in the SHR-N group. The tendency was inverse after TSC treatment, which indicated that TSC may correct the urea metabolism disorder and asparagine level after hypertension, maintaining the body’s steady state.

Tyrosine is a nonessential amino acid that can be synthesized from plant-derived phenylalanine and then used for catecholamine synthesis. Tyrosine hydroxylase (TH), the rate-limiting enzyme in catecholamines biosynthesis, is involved in hypertension development (Congo Carbajosa et al., 2015). Hypertension may be associated with alterations in tyrosine hydroxylase activity (Masserano and Weiner, 1983). Schroeder and Adams found that the intravenous injection of tyrosinase (TYR), a phenolic oxidase obtained from mushrooms, consistently lowers the blood pressure of rats made hypertensive by three different methods, while on the average not affecting the blood pressure of normal animals. They indicated that TYR, an enzyme specific in altering phenolic compounds, effectively combated arterial hypertension (Schroeder and Adams, 1941). It was firstly found to be a target of TSC on treatment of hypertension in our results.

In addition, some other amino acids or enzymes were predicted related to SHR and TSC treatment. For example, several studies have shown that variation in serum aspartate aminotransferase (ALT) activity in the population is associated with risk of development of cardiovascular disease, stroke and hypertension (Whitfield et al., 2002; Nakanishi et al., 2004), while gastrodin and ferulic acid, as the major active ingredients in TSC, were reported to decrease the activity of ALT in serum (Song et al., 2014; Zhao et al., 2015); the findings of Prast et al. showed changes in histamine levels and histidine decarboxylase (HDC) activities in SHR and suggested involvement of histaminergic neurons in hypertension (Prast et al., 1988); endogenous H_2_S is generated from cysteine, and the key enzymes that catalyze this process include cystathionine-γ-synthase (CBS), cystathionine-β-lyase (CSE), and 3-mercaptopyruvate sulfurtransferase (MPST) in the mammal (Kimura, 2011). CBS is highly expressed in the hippocampus and cerebellum and is a predominant H_2_S-generating enzyme in the brain and nervous tissues. Endogenous activation of CBS, showing an increasing H_2_S, could be a therapeutic approach to prevent deleterious vascular remodeling and hypertension (Sen et al., 2010). Cystathionine γ-lyase (CTH) catalyzes the formation and transformations of sulfane sulfur-containing compounds and plays a pivotal role in the L-cysteine desulfuration pathway. CTH knockout mice have decreased levels of H_2_S (Kabil and Banerjee, 2010). Disruption of CTH in mice leads to cardiovascular dysfunction and marked hypertension. It was reported that gastrodin could up-regulate the expression of CBS in liver cells (Bai et al., 2014), while most of the predicted targets were firstly mentioned in the therapeutic role of TSC.

### Oxidative Stress

Glutamate inhibits the glutamate-cystine antiporter system, resulting in a significant decrease in levels of intracellular GSH, accompanied by an increase in reactive oxygen species, and finally leading to the induction of oxidative stress. Oxidative stress is also closely related to the pathophysiology of hypertension, for the imbalance between antioxidants and pro-oxidants is a major factor of vascular impairment, which leads to blood pressure increasing (Touyz, 2004).

MDA is a type of lipid peroxide that is known to further inhibit mitochondrial electron transport systems and oxidize sulfhydryl groups in proteins, thereby altering their function or disrupting signal transduction pathways (de Champlain et al., 2004). Kashyap et al. found that MDA levels in patients with hypertension were higher than those in people with normal blood pressure (Kashyap et al. 2005). Rodrigo et al. (2007)found that patients with essential hypertension had higher levels of erythrocyte malondialdehyde compared with the normal population, and it was even reported that the level of MDA in patients with hypertension was positively correlated with mean arterial pressure. Similarly, our results showed that MDA levels were remarkably higher in SHR-N rats than in controls, and there was a positive effect of TSC on decreasing MDA levels of SHR-N rats.

As the antioxidant enzymes, SOD and GSH-Px are the first line of defense in the cells against oxidative damage. SOD converts superoxide anions into H_2_O_2_, which is the substrate for GSH-Px. GSH-Px reduces both H_2_O_2_ and organic hydroperoxides when meeting and reacting with glutathione (Rodrigo et al., 2013). Our data found that the activities of SOD were lower in SHR-N rats than in normal rats, and the results were inverse after TSC treatment. These observations reflected that TSC might exhibit an effect to reduce antioxidant production, by that means rendering an individual less susceptible to oxidation damage. Ferulic acid, as a scavenger of free radicals, was reported to lower blood pressure, decrease free radical generation and increase SOD and catalase activity (Alam et al., 2013).

In addition, we speculated that some key enzymes may participate in the pathological process of hypertension and treatment of TSC by interfering with neurotransmitter metabolism. Dopa decarboxylase (DDC) is responsible for the synthesis of the key neurotransmitters dopamine and serotonin *via* decarboxylation of L-3,4-dihydroxyphenylalanine (L-DOPA) and L-5-hydroxytryptophan, respectively. DDC has been implicated in a number of clinic disorders, including Parkinson’s disease and hypertension (Burkhard et al., 2001). Suppression of dopaminergic activity by chronic administration of carbidopa, an inhibitor of DDC, accelerated the development of hypertension in SHRs (Yoshimura et al., 1987). Hollon et al. (2002) have reported that a deficiency of the D5 receptor in mice produces arterial hypertension. The hypertension in the D5^-^/^-^ mice is associated with increased phospholipase D (PLD) expression and activity. Impaired D5 receptor regulation of PLD2 may play a role in the pathogenesis of hypertension (Yang et al., 2005). Mice lacking D5 dopamine receptors have increased sympathetic tone and they may be hypertensive. However, the mechanism of these targets in the treatment of hypertension by TSC has not been reported.

Overall, TSC treated SHRs primarily by interfering with energy metabolism, amino acid metabolism and oxidative stress processes, and glucose, LDL/VLDL, alanine, asparagine, glutamate, glutamine, lactate, methionine, myo-Inositol, serine, threonine, tyrosine and valine were finally considered as the biomarkers of TSC on hypertension in this study, which was partly consistent with the published data. Based on these results, we predicted TH, CBS, DDC, CTH, TYR, HDC, PLD2, AGXT2, KAT and ALT as the anti-hypertension targets of TSC, which were the key nodes in the development of hypertension. Most of the targets were firstly involved in TSC treatment, and we would like to validate the targets with several experimental methods from multiple angles in our following study.

## Conclusion

On the basis of the ^1^H NMR-based analytical approach, we have obtained the metabolomics results of SHRs after treatment with TSC. Based on the results, we found that TSC could inhibit the process of glycolysis and energy supply, present obvious relevance with some amino acid metabolism, and disturb some oxidative and antioxidative enzymes, defending against oxidative injury after hypertension. Our findings may help to understand the anti-hypertensive mechanisms of TSC and provide a theoretical basis for its future research, development and clinical applications.

## Ethics Statement

The protocol was approved by the Animal Ethical and Welfare Committee, Beijing University of Chinese Medicine.

## Author Contributions

JG, JC and HZ designed the research, conducted and performed the majority of the experiment, and revised the manuscript. TW, CW, SW, DM and YL assisted and supported several experimental performances and dealt with the statistical data. CW, WW, JG and HZ supervised the research and revised the manuscript.

## Funding

This work was supported by grants from Special Funds of Beijing Municipal Science and Technology Commission in 2016: Study on the Effect of Chaihujialonggumulitang on Coronary Heart Disease with Anxiety Based on Psycho-cardiology (No. Z161100000516136); China Postdoctoral Science Foundation (2018M641286); and the Fundamental Research Funds for the Central Universities (Beijing University of Chinese Medicine: Young Teachers Project) (2019-JYB-JS-163); "(Double First Class) "Guidance Project-Teacher Team Building Project.

## Conflict of Interest Statement

The authors declare that the research was conducted in the absence of any commercial or financial relationships that could be construed as a potential conflict of interest.
